# Novel Molecular Mechanisms Underlying the Ameliorative Effect of Platelet-Rich Plasma against Electron Radiation-Induced Premature Ovarian Failure

**DOI:** 10.3390/ijms251810115

**Published:** 2024-09-20

**Authors:** Grigory Demyashkin, Matvey Vadyukhin, Zaira Murtazalieva, Ekaterina Pugacheva, Vladimir Schekin, Makka Bimurzaeva, Svetlana Pesegova, Petr Shegay, Andrey Kaprin

**Affiliations:** 1 Department of Digital Oncomorphology, National Medical Research Centre of Radiology, 2nd Botkinsky Pass., 3, 125284 Moscow, Russia; vma20@mail.ru (M.V.); dr.shchekin@mail.ru (V.S.); dr.shegai@mail.ru (P.S.); kaprin@mail.ru (A.K.); 2 Laboratory of Histology and Immunohistochemistry, Institute of Translational Medicine and Biotechnology, Sechenov University, Trubetskaya St., 8/2, 119991 Moscow, Russia; zaria.alieva.90@bk.ru (Z.M.); rouzella@mail.ru (E.P.); bimakka@mail.ru (M.B.); 3 Research and Educational Resource Center for Immunophenotyping, Digital Spatial Profiling and Ultrastructural Analysis Innovative Technologies, Peoples’ Friendship University of Russia (RUDN University), Miklouho-Maclay St., 6, 117198 Moscow, Russia; pesegova22@mail.ru; 4 Department of Urology and Operative Nephrology, Peoples’ Friendship University of Russia (RUDN University), Miklouho-Maclay St., 6, 117198 Moscow, Russia

**Keywords:** premature ovarian failure, experimental radiation injuries, fast electrons, growth factors, molecular biology, immunocytochemistry

## Abstract

Radiotherapy is one of the risk factors for radiation-induced premature ovarian failure and infertility in cancer patients. The development of methods for ovarian radioprotection remains relevant. Moreover, electrons are a little-studied and promising method of radiation with the least toxic effect on normal tissues. The assessment of intracellular mechanisms regulating the protective effects of leukocyte-poor platelet-rich plasma in a model of radiation-induced premature ovarian failure caused by electron irradiation. Wistar rats were divided into four groups, namely a control group, irradiation group (electron exposure), irradiation + leukocyte-poor platelet-rich plasma group, and only leukocyte-poor platelet-rich plasma group. Fragments of ovaries were removed and hormonal, oxidant, histological, and morphometric studies were carried out. The cell cycle of ovarian follicles and the inflammatory and vascular response were assessed using immunohistochemistry. The activity of MAPK, ERK, and PI3K pathways was also assessed using the RT-qPCR. We found that electron irradiation causes a decrease in the functional activity of the ovaries and the death of follicular cells through apoptosis. The administration of LP-PRP led to a partial restoration of the cytokine balance. In addition, minor ovarian damage and mild inflammation were observed in this group. Leukocyte-poor platelet-rich plasma components have anti-inflammatory, angiogenetic, and radioprotective effects, reducing the activation of the NOX4, caspase and cytokine cascades, and inflammatory response severity through the MAPK/p38/JNK signaling pathway. This leads to the induction of endogenous antioxidant protection, the repair of post-radiation follicular damage, and slowing down the development of radiation-induced premature ovarian failure after electron irradiation.

## 1. Introduction

The etiology and mechanisms of the development of premature ovarian failure remain unsolved. Radiotherapy for malignant neoplasms of the pelvic region is one of its risk factors [1,2]. Thus, not only atypical ovarian cells are irradiated but also normal oocytes. These cells have a high degree of radiosensitivity due to their high proliferative activity [3]. Then, the death of normal follicles occurs, which leads to the development of radiation-induced premature ovarian failure (RIPOF) and infertility as a late complication. This highlights the urgent need for research into the molecular pathogenetic mechanisms of RIPOF. Moreover, it is important to develop methods for the protection of normal follicles and reproductive function during radiotherapy in cancer patients [4].

The impact of ionizing radiation (IR) at the molecular level occurs through a direct mechanism via DNA breaks or crosslinks, as well as chromosomal aberrations [5]. Another important pathway is the indirect mechanism via the generation of reactive oxygen species and reactive nitrogen species as a result of molecular water radiolysis, as well as lipid peroxidation products [6]. This leads to oxidative stress development and the death of normal oocytes by apoptosis, necroptosis, and mitochondrial-driven necrosis. At the same time, immune cells are attracted by the formation of damage-associated molecular patterns (DAMPs) and the expression of alarmins and cytokines (TGF-β, TNF-α, IL-1, IL-6) [7,8,9].

An important role in inflammation triggering in response to primary cell death through the apoptosis cascade (as a result of the accumulation of unrepaired DNA mutations) is played by the NF-κB, MAPK, ERK, PI3K/Akt, and TNF-α signaling pathways, as well as the activation of the NLRP-3 inflammasomes, etc. [6,10]. Thus, pro-inflammatory (IL-1β, IL-6, IL-8, TNF-α, etc.) or anti-inflammatory (IL-4, IL-10, TGF-β, prostaglandin E2, etc.) imbalance occurs with the development of inflammatory reactions [11]. This leads to the recruitment of macrophages and cellular infiltration [12]. It is likely that the MAPK signaling pathway plays a leading role in the induction of apoptosis of granulosa cells primarily through the internal pathway and caspase-3 is the terminal effector protein.

However, there are no targeted studies devoted to the detection and CD immunotyping of inflammatory cells in the ovaries after exposure to electron irradiation.

It is known that electrons are a little-studied and promising method of radiation therapy with the least toxic effect on normal tissues compared to other types of ionizing radiation (photons, β-, and γ-rays) [13,14]. This makes research devoted to molecular aspects of electron radiotherapy in the ovaries even more relevant.

One of the promising regenerative substrates is leukocyte-poor platelet-rich plasma (LP-PRP), which contains a large number of biologically active substances, such as platelet-derived growth factors, insulin-like growth factors, vascular endothelial growth factors, platelet angiogenic factors, transforming growth factor-β, fibroblast growth factors, epidermal growth factors, connective tissue growth factors, interleukin-8, fibronectin, vitronectin, sphingosine-1-phosphate, etc. [15].

Some authors have suggested that LP-PRP is a promising regenerative substrate for research not only in the field of wound and tissue healing [16] but also in the treatment of radiation-induced damage in several organs, including the ovaries [17,18]. This is probably due to the ability of biologically active molecules in LP-PRP to restore tissue by initiating the proliferation of surviving cells, chemotaxis, extracellular matrix synthesis, remodeling, angiogenesis, epithelization, etc. [15]. Due to the regenerative activity of LP-PRP, it is very interesting to look at the molecular mechanisms of LP-PRP action in the RIPOF model in the experiment.

Thus, it is necessary to conduct research like this in order to determine the dose-dependent effects of electron irradiation on the proliferation and apoptosis of follicles, as well as to assess the risks of developing RIPOF. Such work is also necessary to determine the optimal doses for cancer electron therapy in the pelvic area to level out neighboring organs’ radiation damage. Particularly important is the search for drugs that can improve cancer patients’ prognosis and prevent RIPOF development after radiotherapy in the long term.

The aim of this study is the assessment of intracellular mechanisms regulating the protective effects of leukocyte-poor platelet-rich plasma in a model of radiation-induced premature ovarian failure caused by electron irradiation. For this purpose, the hormonal (FSH, AMH) and oxidative stress (NOX4) levels, a histological assessment, the proliferation and apoptosis of ovarian follicles, and the inflammatory (IL-1β, IL-6, IL-4, IL-10, CD3, CD20) and vascular (VEGF-A) response of interstitial tissue, as well as ERK, MAPK/p38/JNK, and PI3K/Akt pathways, were assessed.

## 2. Results

### 2.1. Hormonal Status Assessment

**Figure 1:**
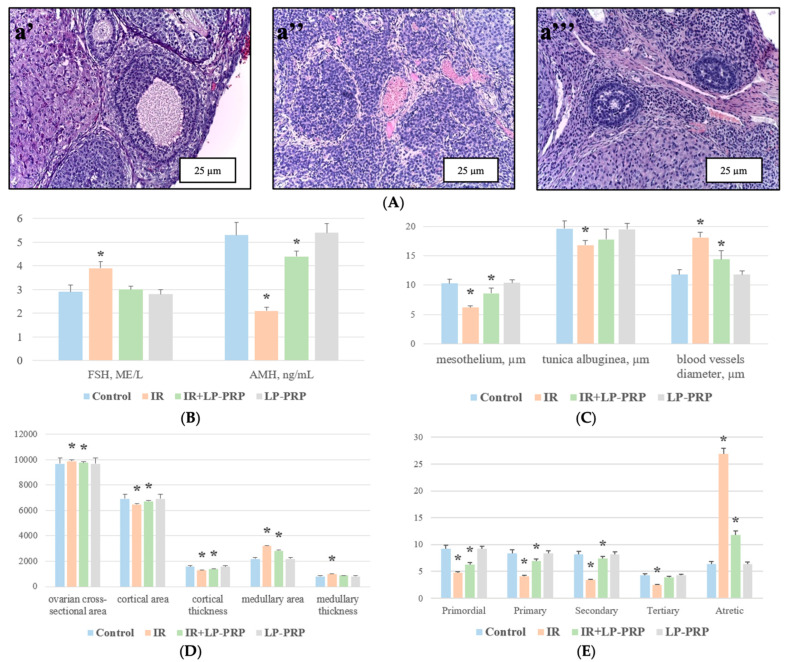
Morphological and morphometric ovarian assessment in the control and experimental groups. (**A**)—ovary fragments on the 7th day of the experiment of the control group (**a’**), irradiation group (**a’’**), and irradiation + LP-PRP group (**a’’’**); hematoxylin and eosin staining, magn. ×200. (**B**)—levels of sex hormones, FSH (in IU/L) and AMH (in ng/mL), in blood plasma of the control group, irradiation group (IR), irradiation + LP-PRP group (IR + LP-PRP), and LP-PRP group. (**C**)—morphometric measurements of the ovarian mesothelium and tunica albuginea thickness and blood vessels’ diameter in the control group, irradiation group (IR), irradiation + LP-PRP group (IR + LP-PRP), and LP-PRP group, in µm. (**D**)—morphometric measurements of the ovarian cross-sectional area (×10^3^ µm^2^), as well as the cortex and medulla area (×10^3^ µm^2^) and thickness (µm), in the ontrol group, irradiation group (IR), irradiation + LP-PRP group (IR + LP-PRP), and LP-PRP group. (**E**)—average number of follicles at different stages of maturation in the control group, irradiation group (IR), irradiation + LP-PRP group (IR + LP-PRP), and LP-PRP group. *—statistically significant difference compared to the control; *p* < 0.05. LP-PRP—leukocyte-poor platelet-rich plasma.

The concentration of sex hormones was determined in the blood serum to assess the ovarian reserve, namely the follicle-stimulating hormone (FSH) in the first phase of the cycle (on days 2–3) and the anti-Mullerian hormone (AMH). After local electron irradiation, an increase in the level of the FSH and a decrease in the level of the AMH were noted and the pre-irradiation administration of LP-PRP to animals of group III showed a trend toward control values (Figure 1B).

### 2.2. Macroscopic Study

The ovaries after electron irradiation were found to be reduced in size, flattened in shape with a coarsely lumpy surface, and whitish-yellow-colored with a thin tunica albuginea, as well as having deep grooves and slight asymmetry during the macroscopic evaluation. The macroscopic examination of the ovaries of groups III and IV did not reveal any visual differences compared to the control.

### 2.3. Microscopic and Morphometric Studies

A normal ovarian structure was observed in the control and IV groups during a microscopic examination on the seventh day of the experiment; the superficially located mesothelium covers the outside of the tunica albuginea (a layer of dense fibrous connective tissue) and in the cortex, there are follicles at different stages of development (Figure 1A). The signs of RIPOF were noted after 20 Gy fractional local electron irradiation. These included a sharp decrease in the proportion of primordial, primary, secondary, and tertiary follicles, along with an increase in the number of atretic follicles. There was also pyknosis observed in some oocytes and partial fragmentation of granulosa cells. Small hemorrhages were present in the stroma, and the thickening of connective tissue cords was noted. Additionally, stasis was observed in the blood vessels’ lumen in the interstitial tissue and rete ovary. In single preserved follicles, there is a decrease in the number of theca cells (Figure 1A).

At the same time, the pre-irradiation administration of leukocyte-poor platelet-rich plasma led to less pronounced radiation-induced histoarchitectural damage in the group III ovaries compared to group II. A slight decrease in the proportion of primordial, primary, secondary, and tertiary follicles was observed in combination with a small number of atretic follicles’ appearance, as well as isolated hemorrhages into the stroma and stasis in the interstitial tissue and rete ovary blood vessels’ lumen (Figure 1A).

A morphometric study of the ovaries after fractional local electron irradiation demonstrated a decrease in the mesothelium and tunica albuginea thickness, as well as the cortex thickness and area, compared to the control. At the same time, an increase in the ovarian cross-sectional area, the medulla area, and thickness, as well as the blood vessels’ diameter, was observed. Changes in these parameters were less pronounced in the pre-radiation LP-PRP administration group (Figure 1C–E).

### 2.4. Cell Cycle Assessment

**Figure 2:**
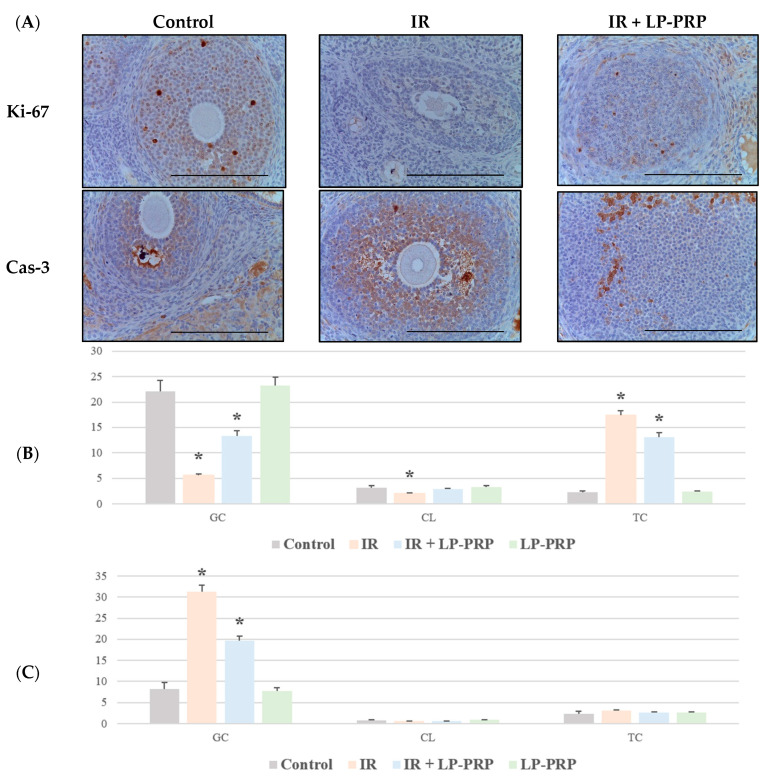
Proliferation and apoptosis in ovaries of the control, irradiated (IR), and experimental (IR + LP-PRP) groups. (**A**)—immunohistochemical reactions with antibodies to Ki-67 (top row) and caspase-3 (bottom row), magn. ×400. (**B**)—distribution of Ki-67-immunopositive cells in the ovaries of the control and experimental groups, graph. (**C**)—distribution of caspase-3-immunopositive cells in the ovaries of the control and experimental groups, graph. The scale bar is 25 µm. GC—granulosa cell, CL—corpus luteum, TC—theca cell, LP-PRP—leukocyte-poor platelet-rich plasma. Data are presented as means ± standard error. *—statistically significant differences compared to the control group (*p* < 0.05).

An IHC study of the ovaries after 20 Gy electron irradiation revealed a decrease in Ki-67 expression in the follicles (3.9 times) and corpus luteum (1.5 times), while the proportion of Ki-67-positive theca cells increased sharply (7.5 times) compared to the control (Figure 2A,B). The number of caspase-3-immunopositive granulosa cells was increased by 3.8 times, while no differences were observed in the corpus luteum and theca compared to the control (Figure 2A,C). Pre-irradiation LP-PRP administration led to a partial restoration of the proliferative activity of granulosa cells (2.4 times) and the proportion of Ki-67-positive theca cells decreased (1.3 times) in group III compared to the irradiation group (Figure 2A,B). A significant decrease (1.6 times) in the caspase-3 expression was observed only in granulosa cells in group III compared to the irradiation group (Figure 2A,C).

### 2.5. Inflammation Assessment

**Figure 3:**
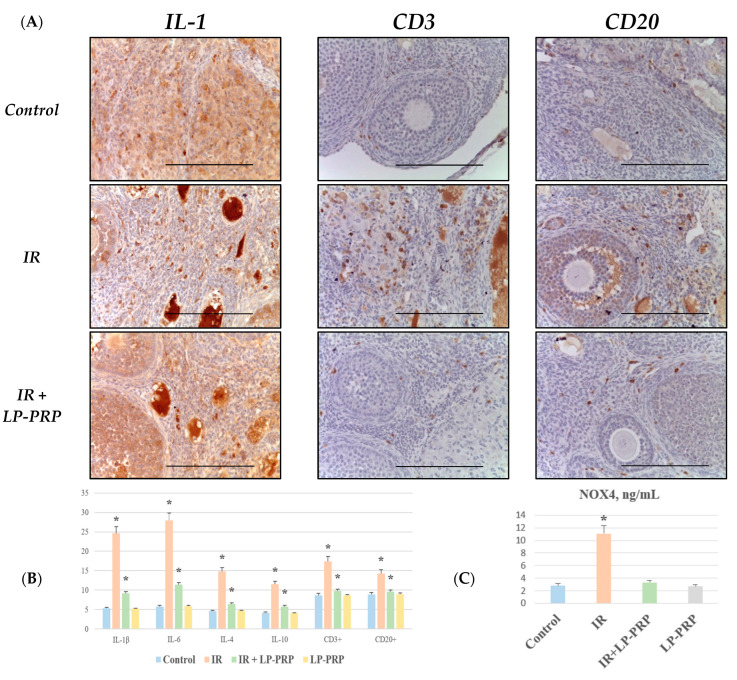
Microphotographs of ovaries in the control, irradiated (IR), and experimental (IR + LP-PRP) groups (**A**). Left—immunohistochemical reactions with antibodies to the pro-inflammatory cytokine IL-1β, magn. ×400; in the center—immunohistochemical reactions with antibodies to CD3, magn. ×400; on the right—immunohistochemical reactions with antibodies to CD20, magn. ×400. The scale bar is 25 µm. Distribution of pro-inflammatory (IL-1β, IL-6) and anti-inflammatory (IL-4, IL-10) cytokines, as well as CD3+ and CD20+ lymphocytes in the ovaries of the control and experimental groups, graph (**B**). NOX4 activity in tissue homogenate using an ELISA, in ng/mL, graph (**C**). LP-PRP—leukocyte-poor platelet-rich plasma. Data are presented as means ± standard error. *—statistically significant differences compared to the control group (*p* < 0.05).

In an IHC study of the expression of pro-inflammatory (IL-1β, IL-6) and anti-inflammatory (IL-4, IL-10) cytokines in the ovaries after 20 Gy local electron irradiation, a sharp increase in the expression of IL-1β (4.6 times) and IL-6 (4.8 times) was noted compared to the control group, while the levels of immunolabeling for IL-4 and IL-10 exceeded control values by only 3.2 times and 2.7 times, respectively (Figure 3). An IHC study of the ovaries with antibodies to T-cells (CD3) and B-cells (CD20) after local electron irradiation revealed both CD3+ and CD20+ immune cells located predominantly perivascularly and their number significantly exceeded the control values by 2.0 times and 1.6 times, respectively (Figure 2). A predominance of CD3+ immunocompetent cells (1.2 times) was noted relative to the proportion of CD20+ lymphocytes in the group II ovaries in a quantitative analysis (Figure 3A,B). It should be noted that electron irradiation led to an increase in the concentration of the oxidative stress marker NOX4 by more than three times compared to the control (Figure 3C).

Pre-irradiation LP-PRP administration led to a less pronounced increase in the expression levels of pro-inflammatory cytokines (1.7 times for IL-1β and 2.0 times for IL-6) compared to the control group and anti-inflammatory cytokines showed almost similar values and exceeded the control values by 1.4 times (IL-4) and 1.4 times (IL-10) (Figure 3). Interestingly, pre-irradiation LP-PRP administration also reduced the severity of CD3+ and CD20+ cellular inflammatory infiltration in the group III ovaries compared to the control by 1.1 times and the proportion of CD3+ lymphocytes exceeded the number of CD20+ lymphocytes (Figure 3A,B).

### 2.6. Angiogenesis Assessment

**Figure 4:**
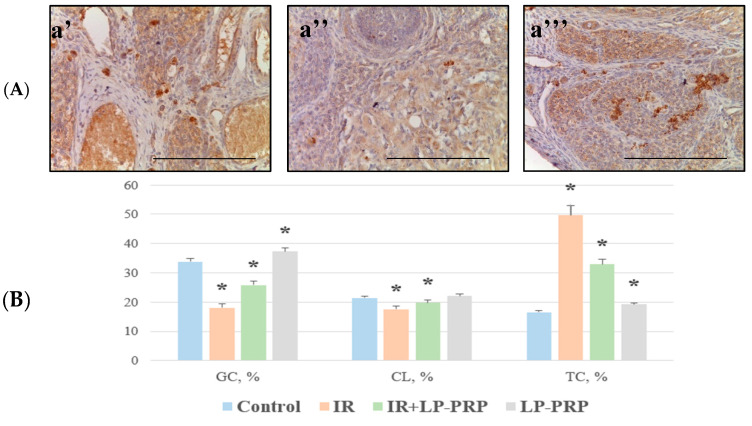
Microphotographs of ovaries in the control (**a’**), irradiated ((**a’’**); IR), and experimental ((**a’’’**); IR + LP-PRP) groups; immunohistochemical reactions with antibodies to VEGF-A, magn. ×400 (**A**). The scale bar is 25 µm. Distribution of VEGF-A in the ovaries of the control and experimental groups, graph (**B**). GC—granulosa cell, CL—corpus luteum, TC—theca cell, LP-PRP—leukocyte-poor platelet-rich plasma. Data are presented as means ± standard error. *—statistically significant differences compared to the control group (*p* < 0.05).

According to the results of an IHC study with antibodies to VEGF-A a week after electron irradiation, a decrease in the proportion of IHC-positive granulosa cells was revealed (1.9 times) compared to the control. The immunolabeling of some cells in the corpus luteum was also observed and its number was 1.2 times lower compared to the control. At the same time, the number of VEGF-positive endothelial cells increased, which is associated with an increase in the number of small- and medium-sized blood vessels per unit area (neovasculogenesis). On the contrary, the proportion of intensely stained VEGF-A-positive theca cells was increased by 3.0 times compared to the control (Figure 4).

Pre-irradiation LP-PRP administration led to the intensification of VEGF-A immunolabeling; an increased number of immunopositive granulosa cells was observed in group III, exceeding the values of group II by 1.4 times. The number of VEGF-A-positive lutein cells and endothelial cells in the corpus luteum was close to the control values. In the theca cells, there was an intense immunolabeling of endocrinocytes, which the proportion of was reduced by 1.5 times compared to the group of irradiated animals (Figure 4).

### 2.7. Molecular Pathways’ Activity

**Figure 5:**
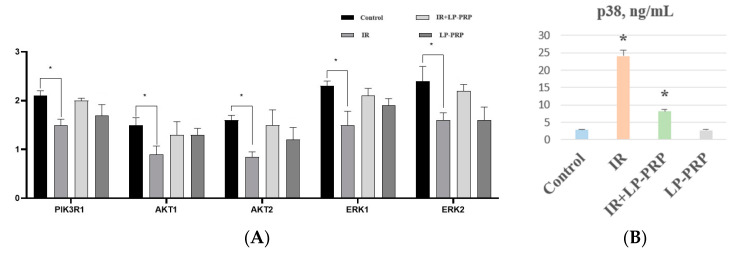
Expression of genes of the PI3K/AKT (*PI3KR1, Akt1, Akt2*), ERK (*Erk1, Erk2*), and MAPK/p38/JNK signaling pathways in the rats’ ovaries in control and seven days after local electron irradiation (IR) and/or leukocyte-poor platelet-rich plasma (LP-PRP) injections (**A**). The abscissa axis shows the studied genes in the control and experimental groups; the ordinate axis shows the relative gene expression. Data are presented as means and confidence intervals. Expression levels of p38 in control and experimental groups by an ELISA in ng/mL, graph (**B**). Data are presented as means ± standard error. *—statistically significant differences compared to the control (*p* < 0.05).

In molecular analysis, we found an increase in PI3K/Akt, MAPK/p38/JNK, and ERK pathways’ gene expression in response to electron exposure. However, the increased activity levels were partially maintained by pre-irradiation LP-PRP administration. Particularly, the predominance of participants in the MAPK/p38/JNK pathway was observed, which was responsible for post-radiation effects in the ovaries both during irradiation (II group) and during the pre-irradiation administration of LP-PRP (III group) (Figure 5).

## 3. Discussion

This study is devoted to the molecular biological assessment of the relationship between key factors regulating the life cycle of and inflammation in the ovaries after LP-PRP administration in a RIPOF model.

Modern therapy with cytostatics and ionizing radiation corpuscular types in relation to pelvic organ cancers causes not only atypical cell molecular mechanisms and metabolomics’ disruption but also radiosensitive normal ovarian oocyte damage [4,19]. Then, oocytes can repair, or death occurs and the dead fragments are eliminated by phagocytosis. This leads to a decrease in the ovarian reserve and infertility as a consequence [20,21].

According to most studies, the AMH is not controlled by gonadotropic hormones (FSH, LH) and its level reflects the proportion of antral follicles with a more pronounced correlation than other hormonal tests that determine the ovarian reserve (basal levels of FSH, LH, inhibin B, and estradiol) [22,23]. Thus, we can talk about a decrease in the functional activity and ovarian reserve after 20 Gy local electron irradiation based on the levels of FSHs and AMHs.

In the present study, signs of RIPOF were revealed according to the results of a microscopic examination. Thus, we can talk about the post-radiation death of follicle cells mediated both directly (generation of DNA mutations) and indirectly with the formation of high concentrations of toxic-free radicals (products of water radiolysis, lipid peroxidation, etc.) and the suppression of endogenous antioxidant defense [4]. Moreover, the death of follicular cells after electron irradiation by apoptosis was confirmed in an IHC study with antibodies to caspase-3.

Based on the results of the fractional electron irradiation group, a sharp decrease in the primordial follicles’ number was revealed, which is likely due to the direct and indirect effects of ionizing radiation on the ovaries, correlating with the duration of exposure [24]. Reactive oxygen species (ROS) were proven to play an important role in growth inhibition and apoptosis activation in cultured mouse antral follicles in a similar study [7]. Other authors have shown that wave types of ionizing radiation can lead to oocyte meiosis arrest with an inhibition of granulosa cell proliferation and an increase in interstitial cells’ pathological proliferation, which can accelerate the aging process of the ovaries with a gradual depletion of their number and lead to a decrease in reproductive function as a consequence [22].

The MAPK/p38/JNK signaling pathway probably plays a key role in the process of cell death in response to NOX4 oxidative stress as in other types of ionizing radiation [25]. We found an increase in the activity of MAPK/p38 and PI3K/Akt pathways that can both be activated in response to increased concentrations of NOX4. This leads to phosphorylation of the downstream proteins AKT (for PI3K) and ERK. It was previously noted that thioredoxin is a key factor that triggers the activation of the MAPK pathway through p38 and JNK, and we found a significant increase in the activity of this pathway both in the irradiation group and with the introduction of LP-PRP. However, we also noted the activation of the PI3K/Akt pathway in response to irradiation in the ovaries, which may also be a consequence of an increase in the level of NOX4.

It is interesting that the histological disorders we identified during 20 Gy electron irradiation were less pronounced compared to other types of ionizing radiation, which indicates a mild toxic effect of the electrons [26]. For example, some authors discovered a sharp decrease in the follicles’ number after γ-rays whole-body irradiation even at a 1 Gy dose [14]. In the same study, the development of post-radiation fibrosis in the stroma was detected after 5 and 10 Gy γ-irradiation. Moreover, only single primordial follicles were identified with their complete absence along the periphery of the ovaries irradiated with 10 Gy. On the contrary, in our study, primordial follicles were preserved, including those along the periphery after fractional electron irradiation even at a 20 Gy summary dose. Thus, γ-irradiation leads to more pronounced ovarian damage compared to local electron irradiation.

In another study, the authors exposed mice to a 4 Gy single dose of X-rays and found an 81% decrease in the primordial follicles’ number. There was no significant difference in the antral follicles’ number in the X-ray irradiation group compared with the control. However, the authors also found a significant increase in the atretic follicles’ number in irradiated ovaries by 300% [13]. Apparently, ovarian electron exposure is safer for normal follicles than X-rays.

Thus, the molecular mechanisms of follicular damage after electron irradiation are in many ways similar to the X-rays and gamma irradiation mechanisms, but β-particles have special physical and chemical characteristics (low mass, charge, etc.), which leads to the modulation of qualitative and quantitative indicators of the activation of the main reactive pathways’ molecules. This results in decreased immune cell recruitment, cytokine production, and a low activation of cytokine cascades. As a consequence, we note a low depth and range of RIPOF after electron exposure. It is very important to note that while being relatively safe in relation to normal tissues, electron irradiation remains highly effective against atypical cells [26].

Radiation-induced cell death most often occurs by apoptosis with activation of the cytochrome *c* and caspase cascade pathways and in our study, granulosa cells turned out to be the most sensitive to electron exposure, which was accompanied by a sharp induction of their apoptosis confirmed by a high level of caspase-3 expression. Almost similar results with other types of ionizing radiation were obtained in another study [27]. In addition, it is possible that the disruption of the secretory function of these cells is accompanied by a decrease in the synthesis of steroid hormones, growth factors, and other biologically active molecules, followed by a secondary ovofolliculogenesis impairment. In our opinion, the increased Ki-67 immunoreactivity in theca cells found in the present study may be associated with hyperplasia of these cells in response to a decrease in the endocrine function of follicular cells, which is an adaptive response of theca cells to maintain levels of steroid hormones (primarily estrogen) in blood plasma.

The accumulation of damage-associated molecular patterns, or DAMPs (HMGB1, uric acid, heat shock proteins, etc., as a result of the appearance of cell apoptotic products, toxic radicals, mutant genetic material, etc.), in the ovaries leads to the high expression of pro- and anti-inflammatory cytokines [28]. Their increase was confirmed by the results of our IHC study with antibodies to IL-1β, IL-6, IL-4, and IL-10 and in group II, pro-inflammatory cytokines predominated, which probably indicates a cytokine imbalance with an exponential release of pro-inflammatory factors and the depletion of the local anti-inflammatory defense system. Similar data were obtained in the works of other researchers [14,29,30].

In addition, we found an increase in the IHC reaction with antibodies to VEGF-A, which is a marker of angiogenesis and has a direct connection with the MAPK signaling pathway through JNK [25]. In the family of vascular endothelial growth factors, it is customary to distinguish VEGF-A, VEGF-B, VEGF-C, VEGF-D, and VEGF-E, which serve as ligands for transmembrane receptors VEGFR-1, -2, and -3, expressed also by granulosa cells [31]. The VEGF biological effect occurs after binding to the receptor as a result of autophosphorylation and activation of the PI3K/AKT and Ras/MAPK pathways [32]. This leads to the induction of neoangiogenesis and an increase in the blood vessels’ density in the field of view, which we have detected during a microscopic examination. This is probably due to the improvement of biochemical and metabolic processes in the follicles caused by endothelium fenestration and an increased blood—ovarian barrier permeability, which facilitates the entry of other biologically active molecules and regenerative substrates (including LP-PRP components) into the follicular cells. An IHC study confirmed the high expression of VEGF in follicular cells and theca cells, which makes it more likely that the growth factors cascades are activated, and all of the above cellular mechanisms are triggered in ovaries.

Thus, the production of a large number of cytokines in combination with a vascular reaction in response to electron irradiation leads to the migration of T- and B-immune cells into the ovarian stroma with cellular inflammatory infiltration development, which we discovered during an IHC study with antibodies to CD3 and CD20. Interestingly, in groups II and III, we noted a significant increase in the proportion of CD3+-immune cells compared to the number of CD20+ lymphocytes. This indicates the predominance of T-cell immunity over B-cell immunity in ovaries after local electron irradiation. In further studies, it is possible to conduct detailed CD typing of immune cells in order to determine the specific types responsible for ovarian inflammation in response to ionizing radiation exposure.

Due to the fact that ionizing radiation exposure is accompanied by a decrease in the synthesis of key growth factors responsible for the restoration processes in ovarian structures, it was advisable to use leukocyte-poor platelet-rich plasma, in which platelet α-granules contain high concentrations of biologically active molecules capable of inducing the follicular cells’ regenerative activity and metabolism (by activation of neoangiogenesis) [33,34]. Therefore, the most important growth factors in the LP-PRP are insulin-like growth factor-1, transforming growth factor-β, platelet-derived growth factors, vascular endothelial growth factors, etc. [35].

These substances are capable of inducing the proliferation and differentiation of many cell types, thereby restoring the proliferative–apoptotic balance, which was probably responsible for the positive effect of LP-PRP in the present study; higher levels of granulosa cells’ proliferative activity were associated with significantly lower rates of caspase-3 immunoreactivity compared to the irradiation group. Moreover, the pre-irradiation administration of LP-PRP led to a slight increase in the theca cells’ proliferative activity. This may indirectly indicate the body’s low need for compensatory-adaptive theca hyperplasia and its need to keep the steroid hormones close to physiological levels.

An increase in the VEGF-A expression in the corpus luteum vascular endothelium per unit area is associated with increased vascularization (neovascularization), which was of a compensatory-adaptive nature in irradiated animals. The preservation of the VEGF-positive cells’ number in the corpus luteum being close to control values is due to additional angiogenesis factors’ potentiation after LP-PRP administration. However, VEGF was practically not expressed directly in lutein cells, which probably explains its low immunolabeling values in the corpus luteum as a polymorphic structure. As a result of the VEGF effect in ovaries, the positive effects of LP-PRP growth factors are potentiated, including regeneration, which are necessary for adequate and rapid tissue restoration in response to fractional electron irradiation.

At the same time, in the pre-radiation LP-PRP group, a lower degree of inflammatory reaction was noted compared to group II one week after the last fraction. This is confirmed by the pro- and anti-inflammatory cytokine expression levels, as well as markers of T- and B-cell immunity with the predominance of the first, as in group II.

It is interesting that the levels of pro-inflammatory cytokines were close to the expression values of anti-inflammatory cytokines during an IHC study in group III, which indicates the preservation of the cytokine balance and perhaps allows us to indirectly speak about the radioprotective effects of LP-PRP, including its anti-inflammatory effect in ovaries. This is probably due to the large number of biologically active substances in its composition, which have both antioxidant and anti-inflammatory effects [36,37]. The first is to reduce the degree of radiation-induced oxidative stress, the generation of toxic radicals, and the prevention of cell apoptosis (consequently reducing the amount of DAMPs as one of the key inducers of inflammation); the second has been proven in numerous studies and is combined with the induction of repair mechanisms in many tissues [16,38,39].

**Figure 6:**
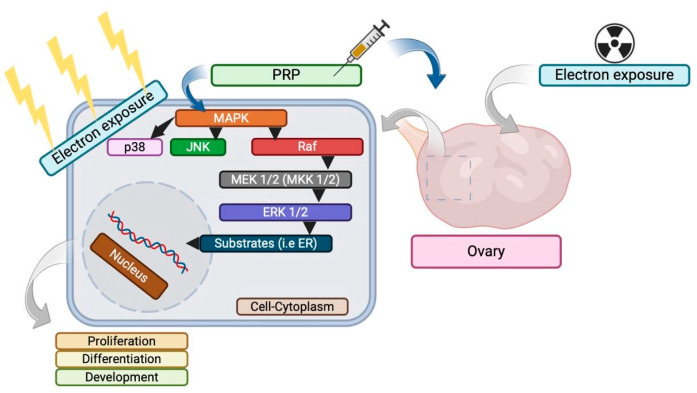
Molecular intracellular mechanisms of action of leukocyte-poor platelet-rich plasma (LP-PRP) in an ovary after local electron exposure, depicted schematically.

Moreover, the findings demonstrated the partial preservation of MAPK/p38/JNK pathway activity, which may be associated with its activation not only during radiation damage to the ovaries but also during ovarian repair using growth factors. The mechanisms of action of LP-PRP via the MAPK pathway are schematically shown in Figure 6. At the same time, the activity of the PI3K/Akt pathway did not differ from the control, which indicates the absence of its participation in ovarian repair processes. However, the use of LP-PRP requires new studies to determine the specific roles of each growth factor in the reparative activity of LP-PRP. Thus, the listed molecular mechanisms of LP-PRP action may indicate its high efficiency in protecting follicular cells from electron exposure, as well as the induction of reparative processes in ovaries. This leads to less pronounced granulosa cell damage and ovarian reserve preservation, as well as reproductive function protection. This was confirmed by the histological study, which found slight changes in the ovarian structures.

However, the study has some limitations, such as a small sample size and a lack of recording of late effects. Nevertheless, summing up the results of the histological, IHC, and molecular studies, it was demonstrated that cell death by apoptosis and local inflammation were triggered in response to electron exposure in ovaries. Pre-radiation LP-PRP administration reduces the electrons’ toxic effect by inducing local antioxidant defenses, angiogenesis, and reparative mechanisms in ovaries. This may indicate the radioprotective (anti-inflammatory and antioxidant) effects of LP-PRP, which may be useful in the prevention of RIPOF and infertility in cancer patients.

## 4. Materials and Methods

### 4.1. Experimental Animals

Wistar rats were used in the experimental study; age 8–9 weeks; n = 120. The animals were kept under a 12-h daylight period, with air conditioning at a temperature of 23 °C and a humidity of 40–60%, and on a standard diet with water ad libitum. The rats were kept in plastic cages with a layer covered with an absorbent substance (rice husk) to provide nesting material. During the long experiment, the animals were placed in cages of two individuals in order to exclude the possible influence of long-term solitary confinement on behavior.

The animals were divided into four experimental groups.

Group I (*n* = 10)—control;

Group II (*n* = 10)—fractional local electron irradiation (total dose 20 Gy);

Group III (*n* = 10)—intraperitoneal administration of leukocyte-poor platelet-rich plasma (LP-PRP) 1 h before fractional local electron irradiation (total dose 20 Gy);

Group IV (*n* = 10)—intraperitoneal administration of LP-PRP.

Animals of all groups were removed from the experiment by high doses of anesthetic (ketamine + xylazine), administered on the 7th day of the experiment (the experiment start date was considered the last day of irradiation).

### 4.2. RIPOF Model

Animals were fractionally locally irradiated with electrons in the pelvic segment (total dose 20 Gy, dose rate 1 Gy/min, energy 10 MeV and frequency 9 Hz, field size Ø 50 mm) using a linear accelerator (“NOVAC-11”, Radiological Department of the experimental sector of the MRRC named after A.F. Tsyba, Obninsk, Russia).

### 4.3. Leukocyte-Poor Platelet-Rich Plasma (LP-PRP)

Blood was taken from the peripheral tail vein and mixed with an anticoagulant (5% sodium citrate solution) based on 10 mL of blood and 1 mL of Na_3_C_6_H_5_O_7_. LP-PRP was obtained by two-stage centrifugation of citrated blood at room temperature (20–24 °C). In the first stage, the centrifugation was at 1800 rpm (≈543.35 g) for 10 min. The resulting supernatant layer of liquid was taken with a syringe and transferred into a clean dry test tube; the second stage is the centrifugation of the supernatant at a speed of 3400 rpm (≈1938.61 g) for 10 min. The supernatant layer was removed and the platelet layer remaining at the bottom was activated with calcium chloride (about 0.05 mL of 10% CaCl_2_). The XT-1600i system was used to analyze the number of blood platelets. The LP-PRP platelet count (1,900,000 platelets/microliter) was approximately 3.0 times that of the blood platelets (610,000 platelets/microliter). Each injection contained approximately 2400 μL of LP-PRP, which was administered immediately after preparation.

### 4.4. Hormonal Status Assessment

Serum levels of follicle-stimulating hormones (FSHs) and anti-Mullerian hormones (AMHs) were determined by spectrophotometry according to the manufacturer’s instructions (FSH ELISA kit, Taisiteng Biotechnology Co., Ltd., Guiyang, China) (Assay kit AMH, ELISA, Cusabio Biotech Co., Wuhan, China). FSH and AMH levels in the samples were calculated from standard curves using the linear regression method and expressed in IU/L and ng/mL, respectively.

### 4.5. Enzyme-Linked Immunosorbent Assay (ELISA)

The MAPK/p38/JNK pathway as well as NOX4 activity studies were performed in ovary homogenates using commercial ELISA kits on rat NOX4 (Cloud-Clone Corp., Houston, TX, USA, Cat. No SEB924Ra) and rat p38 (FineTest, FineTest Biotech Inc., Boulder, CO, USA; Cat. No ER1138) levels. NOX4 and p38 levels in the samples were calculated from standard curves using the linear regression method and expressed in ng/mL.

### 4.6. Reverse Transcription Quantitative Polymerase Chain Reaction (RT-qPCR)

The expression of the main members of the ERK signaling pathway (ERK1, ERK2) and the PI3K/AKT signaling pathway (PIK3R1, AKT1, AKT2) was assessed using RT-qPCR. We extracted cDNA for all the samples. Testis fragments were placed in a stabilizing solution and stored at −70 °C until testing. Samples were homogenized according to the standard protocol. Total RNA extraction was carried out using a set of ready-made reagents using the RNeasy Plus Mini Kit (QIAGEN, Dutch, The Netherlands). Complementary DNA (cDNA) synthesis was performed using Super Script™ VILO™ Master Mix (Invitrogen, Carlsbad, CA, USA). The isolated cDNAs were subjected to a RT-qPCR using a ready-made mixture of ABsolute Blue QPCR Mix reagents (Thermo Scientific, Waltham, MA, USA) with SYBR Green I. The RT-qPCR was carried out using the Step One System (Applied Biosystems, Waltham, MA, USA) and standard software. Gene expression analysis was performed using the threshold cycle (Ct) method and calculating relative gene expression according to the protocol. Control was performed against the housekeeping reference gene GAPDH. The selection of primers was carried out based on publicly available materials on DNA and mRNA sequences of genes in the NCBI database using Primer-BLAST (Table 1).

### 4.7. Morphological Study

The appearance and condition of the parenchyma were assessed after the extraction of the ovaries, which were weighed (in grams) and measured. Then, they were cut parallel to the sagittal plane, fixed in formaldehyde, and after processing (tissue histological processing apparatus, Leica Biosystems, Nussloch, Germany), they were embedded in paraffin blocks from which serial sections were prepared (3 µm thick), deparaffinized, dehydrated, and stained with hematoxylin and eosin for histological examination in accordance with standard protocols.

A morphological analysis was carried out in 10 randomly selected fields of view of the microscope at a magnification of ×400 in 5 random sections from each sample. Digital images of histological sections (scanned preparations) for morphometric studies were obtained using a video microscopy system (Leica DM3000 microscope, Leica Biosystems, Nussloch, Germany; DFC450 C camera; Platrun LG computer) and software for image processing and analysis, Leica Application Suite (LAS) Version 4.9.0.

### 4.8. Morphometric Analysis

Morphometric analysis was carried out using Image J. The following parameters were calculated in each field: number of follicles of different degrees of maturity, mesothelium and tunica albuginea thickness, medulla blood vessel diameter, ovarian cross-sectional area, and cortex and medulla thickness and area. Established morphological criteria were used to classify and count normal follicles. Follicles were counted based on the oocyte nucleus, and the stage of follicle development was determined using standard methods [40]. The integrity of the basement membrane and oocytes was visually assessed.

### 4.9. Immunohistochemical Study

Samples were prepared for IHC analysis in accordance with standard protocols. Monoclonal antibodies to the proliferation factor Ki-67 (ThermoFisher, Waltham, MA, USA, Clone MM1) and the apoptosis termination factor caspase-3 (ThermoFisher, Waltham, USA, Clone 74T2) were used as primary antibodies to assess proliferation and apoptosis in the ovaries. In addition, rabbit polyclonal antibodies to VEGF-A (Millipore, Burlington, MA, USA; clone ABS82; 1:100) were used as primary antibodies to assess angiogenesis. Cell nuclei were counterstained with Mayer’s hematoxylin. The number of immunopositive cells was counted in 10 randomly selected fields of view at a magnification of ×400 (in %).

Polyclonal antibodies to interleukin-1β (IL-1β; ThermoFisher, 1:100, Waltham, MA, USA), interleukin-4 (IL-4; ThermoFisher, 1:100, Waltham, MA, USA), interleukin-6 (IL-6; ThermoFisher, 1:100, Waltham, MA, USA), interleukin-10 (IL-10; ThermoFisher, 1:100, Waltham, MA, USA), as well as ready-to-use polyclonal antibodies (RTU; Leica, Nussloch, Germany) to CD3 and CD20, were used to assess the inflammatory response. Cell nuclei were counterstained with Mayer’s hematoxylin. The number of immunopositive cells was counted in 10 randomly selected fields of view at a magnification of ×400 (the ratio of the number of stained cells to the number of all interstitial tissue cells in the field of view × 100%).

A universal two-component detection system, HiDef Detection™ HRP Polymer system, (Cell Marque, Rocklin, CA, USA), mouse/rabbit anti-IGG, horseradish peroxidase (HRP), and a DAB substrate were used to determine secondary antibodies.

### 4.10. Statistical Analysis

The data obtained as a result of the calculation were processed using the computer program SPSS 12 for Windows (IBM Analytics, Armonk, NY, USA). Data are expressed as mean ± standard error (SE). The Shapiro–Wilk test was used to assess the normality of distribution. When comparing study groups with a distribution other than normal, the Kruskal–Wallis test with Dunn’s post hoc test was used. Multiple comparisons were performed using the Mann–Whitney *U*-test. A *p*-value ≤ 0.05 was considered statistically significant.

## 5. Conclusions

Our study found that leukocyte-poor platelet-rich plasma components have anti-inflammatory, angiogenetic, and radioprotective effects, reducing the activation of the NOX4, caspase and cytokine cascades, and inflammatory response severity through the MAPK/p38/JNK signaling pathway. This leads to the induction of endogenous antioxidant protection, the repair of post-radiation follicular damage, and slowing down the development of radiation-induced premature ovarian failure after electron irradiation.

## Figures and Tables

**Table 1:** Reverse transcription quantitative polymerase chain reaction (RT-qPCR) primer sequences.

Gene	Direct Primer	Reverse Primer
*PIK3R1*	CCCTCAGTGGACTTGGATGT	GCTGCTGGGAATCTGAAAAG
*AKT1*	ACTCATTCCAGACCCACGAC	TGAGCTCGAACAGCTTCTCA
*AKT2*	ATGTAGACTCTCCAGATGAG	TGAGATAATCGAAGTCATTCA
*ERK1*	TCCCAAATCTGACTCCAAAGC	GCCACTGGTTCATCTGTCGG
*ERK2*	GGTTGTTCCCAAACGCTGAC	AATGGGCTCATCACTTGGGT
*GAPDH*	CCGTCTAGAAAAACCTGCC	AGCCAAATTCGTTGTCATACC

## Data Availability

The study did not generate publicly available archival data.
